# The expensive-tissue hypothesis may help explain brain-size reduction during domestication

**DOI:** 10.1080/19420889.2022.2101196

**Published:** 2022-08-05

**Authors:** Raffaela Lesch, Kurt Kotrschal, Andrew C. Kitchener, W. Tecumseh Fitch, Alexander Kotrschal

**Affiliations:** a Institute of Animal Welfare Science, University for Veterinary Medicine, Austria; b Department of Behavioral and Cognitive Biology, Faculty of Life Sciences, University of Vienna, Vienna, Austria; c Department Natural Sciences, National Museums Scotland, Edinburgh, UK; d Behavioural Ecology, Animal Science, Wageningen University and Research, Wageningen, Netherlands

**Keywords:** Brain size, cranial volume, neural crest, gut, intestine

## Abstract

Morphological traits, such as white patches, floppy ears and curly tails, are ubiquitous in domestic animals and are referred to as the ‘domestication syndrome’. A commonly discussed hypothesis that has the potential to provide a unifying explanation for these traits is the ‘neural crest/domestication syndrome hypothesis’. Although this hypothesis has the potential to explain most traits of the domestication syndrome, it only has an indirect connection to the reduction of brain size, which is a typical trait of domestic animals. We discuss how the expensive-tissue hypothesis might help explain brain-size reduction in domestication.

Domestic animals typically have relatively smaller brains than their wild ancestors [1,2]. Together with several morphological traits like white patches, floppy ears and curly tails, these changes are referred to as the ‘domestication syndrome’ [3,4]. A commonly discussed hypothesis, with the potential to provide a unifying explanation for traits captured in the domestication syndrome, is the ‘neural crest/domestication syndrome hypothesis’ [4–6]. It suggests that minor deficiencies in the migration and proliferation of neural crest cells underlie trait formation in domestic animals. Recent comparative work on neural crest genes in domestic and wild animals by Rubio et al. [7] provides support for this hypothesis. A reduced number of neural crest cells arriving at their target sites is directly reflected in the structures that depend on these cells (6]. Therefore, the neural crest/domestication syndrome hypothesis provides clear predictions regarding anatomical/morphological changes, such as a reduction in snout length; a prediction we recently tested in cats [8]. Contrary to this prediction, we found no evidence for a reduction in snout length. This suggests that the neural crest/domestication syndrome hypothesis is not sufficient to explain all trait changes during cat domestication.

A well-documented and nearly ubiquitous trait across domestic animals is a significant reduction in brain size [1], which also occurs in cats [8–10]. While the (cranial) neural crest does have a connection to the development of the fore- and midbrain, the causal connection to brain size itself remains hypothetical [6,11]. Are there other mechanisms that might help explain the documented reduction in brain size?

We suggest that the ‘expensive-tissue hypothesis’ [ETH; 12] provides an alternative and/or complementary explanation for the reduction in brain size seen during cat domestication. This hypothesis originally suggested an energetic trade-off between costly organ systems in the development of humans and other primates. As a classic trade-off, under finite resources, preferred investment in one organ will lead to a reduced investment in other organs [13]. Specifically, the ETH was used to explain variation in primate brain and gut size by suggesting trade-offs between those organs. Since then, numerous comparative studies have corroborated this idea of trade-offs between energetically costly organ systems, from cichlid parental investment to bird flight [14,15]. Artificial selection on brain size over several generations in guppies (*Poecilia reticulata*) yielded causal evidence for a gut-size – brain-size trade-off, since the selection for larger brains was accompanied by shorter guts [16]. We suggest a direct connection between brain-size reduction and gut length in cats (Figure 1).

**Figure 1.**
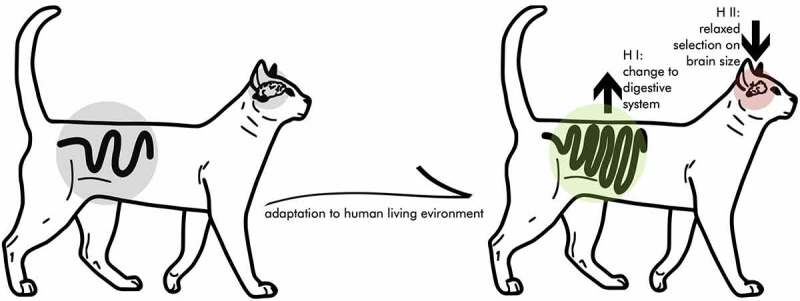
Graphic visualization (and exaggeration) of the expensive-tissue hypothesis in cat domestication. The process of adapting to human environments might have led to a trade-off between brain volume and gut length. This potentially could be explained by two mutually non-exclusive processes outlined in hypothesis I and II.

Two mutually non-exclusive processes could explain a trade-off between brain size and gut length in cats: I) an adaptation of the digestive system to human environments, and II) a relaxation of selection pressures on brain size.

- To benefit from the resources available in human environments (both rodents and refuse/food provided) domestic cats might have adapted toward a more starch-rich diet within the constraints of mainly being obligate carnivores. While cats show genetic adaptations to a hypercarnivorous diet (coinciding with a short digestive tract), they can still digest starch [17–20]. For instance, like dogs, cats’ intestines contain amylase needed for catalyzing the hydrolysis from starch into sugars [21,22]. In fact, one of the most prominent physiological adaptations of dogs in response to a human environment is that they adapted their digestive systems to better process starch-rich food [i.e., leftovers like bread; 21]. To digest diets containing carbohydrates more easily, domestic cats may have experienced selection pressures for increased gut size/length. This increased investment in the digestive system could have made it necessary to divert energy from the brain, thus explaining the relatively smaller brains in domestic cats, compared to wildcats.
- The human environment and readily available food, i.e., rodents attracted by human trash heaps and food storage, might have relaxed selection pressure on brain size. Smaller brains need less energy, which would allow for surplus energy to be invested into other systems like the digestive system (i.e., longer guts).

Recent developments in microbiome research indicate that the change in diet typically associated with domestication leads to profound changes in gut microbiome, which can affect neural development [23]. Considering the ETH, we predict that increased investment in gut tissue would increase the gut’s maintenance costs, which in turn, would be compensated by a reduction in brain size or vice versa. To fully test the relevance of the ETH for domestication, a direct comparison of gut length and brain size across wild cats and domestic cats is necessary.
